# Lost to follow up rate in the first year of ART in adults initiated in a universal test and treat programme: a retrospective cohort study in Ekurhuleni District, South Africa

**DOI:** 10.11604/pamj.2020.37.198.25294

**Published:** 2020-10-29

**Authors:** Patricia Chauke, Mmampedi Huma, Sphiwe Madiba

**Affiliations:** 1 Department of Public Health, School of Health Care Sciences, Sefako Makgatho Health Sciences University, Pretoria, South Africa

**Keywords:** South Africa, universal test and treat, lost to follow up, HIV, retention in care, ART

## Abstract

**Introduction:**

South Africa adopted and implemented the Universal Test and Treat (UTT) strategy for HIV since 2016. However, the care outcomes for patients initiated antiretroviral therapy (ART) through the UTT strategy have not been established. We determined the rate of lost to follow up (LTFU) and associated factors in patients who were initiated on ART through the UTT and the pre-ART strategy at 12 months post ART initiation.

**Methods:**

this retrospective study analyzed the records of a cohort of patients at 12 months post the initiation of ART. We extracted data from the TIER.Net electronic database of selected facilities in a sub-district in Gauteng Province, South Africa. Factors associated with LFTU at 12 months of ART were assessed and logistic regression performed to identify predictors of LFTU.

**Results:**

records of 367 patients were evaluated, and 54% were initiated ART through the UTT strategy. The mean age was 36.3 years, mean CD4 cell count at ART initiation was 341 cells/mm^3^, and 25% were initiated at CD4 cell count above 500 cells/mm^3^. LTFU at 12 months was 28%, 50% were LFTU within six months, and 28% within three months of ART. LFTU in the UTT cohort was higher than in the pre-ART cohort, patients initiated through UTT were twice more likely to be LTFU (AOR = 1.84, CI: 1.13-3.00) than pre-ART patients.

**Conclusion:**

the rate of LTFU at 12 months of ART was 28%, which indicate that the retention in care rate (60%) falls far short of the triple 90 targets required for viral suppression.

## Introduction

Increased access to antiretroviral therapy (ART) in resource-limited settings is an exceptional success story for HIV programs. As of 2017, approximately 21.7 million people were accessing ART globally. An estimated 12.9 million of people living with HIV (PLHIV) in eastern and southern Africa were accessing ART [1]. South Africa accounts for 20% of PLHIV who are on ART globally. Approximately 7.9 million people of all ages were living with HIV in 2017, amongst all PLHIV aged 15 to 64 years who knew their HIV status, 70.6% were on ART [2].

The Universal Test and Treat triple 90 targets require that 90% of all PLHIV know their status, 90% of all people diagnosed with HIV receive sustained ART, and 90% of all people receiving ART are retained in care and have durable viral suppression [1]. Early initiation of ART improve uptake and linkage to care, reduce the severity of HIV morbidity, reduce mortality, slow progression of the disease significantly, and decrease HIV infectivity [3-6]. To reduce mortality and infectivity, patients need to remain in care and actively attend and participate in ART care programs to receive medication and to have their HIV clinical indicators monitored at proper frequencies [4,7].

However, the successes especially that 90% of HIV-infected people will receive sustained ART and 90% will have viral load suppression are being challenged by high rates of loss to follow-up (LTFU) which is a major public health concern [8]. Evidence suggests that 5-year retention in sub-Saharan Africa (SSA) is close to 60% [9-11]. The patient is considered lost if they are 28 up to 180 days late for a scheduled visit consultation or medication pick-up [11,12]. The adoption of 180 days since the last clinic visit has been recommended and adopted as a standard LTFU definition [13].

LTFU reported in a systematic review from studies conducted under earlier treatment guidelines in SSA [6,12] ranges from 23-88% [14]. Estimates of retention in care in SSA are widely variable and the estimated regional average 12-month retention of 76% (range 65% - 89%) is insufficient to achieve the 90-90-90 targets to prevent new HIV infections [9]. The retention in care rate seems to have stayed constant despite the changes in the treatment guidelines. An earlier study conducted in three countries in SSA, found that only 80% of patients started on ART were still in care after one year [11]. However, data from individual studies show a different picture, for example, in a study conducted in Ethiopia only 54% of patients were retained in care at 12 months. Almost 46% of the LTFU had occurred in the first year of ART [15]. Likewise, Brown *et al*. [16] found patient retention in care rate of (61%) in East Africa. In South Africa, pre-ART early retention and LTFU among PLHIV have been consistently estimated at 20%-30% of patients [17,18].

Lost to follow up leads to increased risk of treatment failure, viral rebound, and drug resistance [8]. To reduce mortality and infectivity, patients need to remain in care [7,19]. Retention in HIV care is defined as the continued engagement in health services, from enrollment in care to discharge or death of an individual living with HIV [20]. This is critical to achieve viral load suppression, which is the basis of individual clinical benefits and has the potential to stop the HIV epidemic on a public health level [3,21]. Failure to achieve and sustain viral load suppression poses increased risk of HIV transmissibility [21,22].

Individual factors associated with LTFU remain complex and inconsistent across different settings. While research has shown that sociodemographic and economic factors may be associated with ART initiation in SSA [10,23], age, male gender, race, education, and household income are associated with LTFU [7,24-27]. Health system factors influencing LFTU include long distance to health facilities, clinic size, long waiting time, lack of skilled health professionals, and availability of services [28-30]. Clinical characteristics associated with LTFU include advanced WHO clinical stage, a higher CD4 cell count, absence of an AIDS diagnosis, and detectable viral load. Patients in the highest CD4 cells count category and those in the lowest category at ART initiation have higher risk of LTFU in the first year of ART enrolment [8,25,26,31].

As countries adopt and integrate the UTT strategy into their existing treatment frameworks, the problem of LTFU among individuals initiating ART with a higher CD4 count will increase [32-34]. Research shows that initiation of ART on the same day of testing HIV positive is associated with an elevated risk of LTFU in the initial months of starting ART [34]. Consistent with other countries [35], South Africa has implemented the UTT strategy since 2016 [36]. However, there are limited data from South Africa and SSA describing HIV care outcomes after implementation of the UTT strategy [37]. Previous studies conducted before the introduction of the UTT strategy cannot serve as indicators for LFTU in the context of UTT.

In this study, we used routine clinic data to determine the rate of LTFU and associated factors at 12 months of ART in adult patients following the implementation of the UTT strategy. A secondary objective of this study was to determine the proportion of patients who initiated ART through the UTT strategy since patients who were on the pre-ART register were initiated on ART during this period. The pre-ART patients are HIV-positive individuals who had not yet started ART, but were kept in registers because they were not eligible for ART under the guidelines that recommended initiation of ART at CD4 count below 500 cells/μl. The pre-ART patients were not eligible for ART in line with the 2013 National ART guidelines. It is important to understand the risk factors for LTFU to develop support and monitoring strategies to retain patients in care to meet the South African Government´s target of reaching a retention rate of 90% at 12 months of ART [36].

## Methods

### Study design, setting and population

This was a retrospective cohort study design that used the records of a cohort of patients that were initiated on ART in the month of November 2016. Initiation of patients on ART through the UTT strategy was introduced in September 2016, at the same time patients who were on the pre-ART list were also initiated on ART. Therefore, the study population consisted of a cohort of UTT and pre-ART patients who were initiated ART in the month of November 2016. The facilities use the TIER.Net electronic register to capture the files of patients that are initiated on ART and continuously update for follow-up visits. The facilities provide free ART services to PLHIV using the National Department of Health and the WHO guidelines. The patients are managed by the counsellors for adherence counselling, and by nurses/doctors for clinical issues.

The study setting was six randomly selected primary health care (PHC) facilities located in Ekurhuleni North sub-district in Gauteng Province, South Africa. The sub-district has 28 PHC facilities. The study population consisted of the records of naïve adult patients aged 18 years and older that were initiated on ART during the study period. Data from the District Health Information System (DHIS) showed that 1412 patients were initiated on ART in November 2016. At the onset of the UTT program, the number of patients initiated per facility ranged from 13 to 119 per month. A sample size of 367 records was calculated from a population size of 1412 records of patients initiated during the study period in the selected health facilities. The sample size was calculated using the Raosoft sample size calculator [38]. The sample was selected, with a confidence level of 95%, margin of error of 5% and response distribution of 50%. The files of pregnant women, children below the age of 15 years, and those that were reinitiated on ART, were excluded. The analysis included 367 records from the six selected facilities.

### Study variable and definitions

The patients were classified as initiated through the UTT strategy if they were initiated on the same day of positive HIV test results and up to 14 days after the diagnosis. Eligible patients were initiated on first line ART, which is fixed dose combination of Tenofovir (TDF), Emtricitabine (FTC), and Efavirenz (EFV) (1TFE) and follow-up visits were scheduled as per the guidelines. In South Africa LTFU is defined as patients who have not visited the clinic and have not received drugs for more than 90 days after their last clinic visit. LFTU by definition includes patients that are without documentation of transfer, referral, or reported death. Retention on ART, is when a patient is alive and on ART by the end of the 12-months follow-up period.

While we acknowledge that the viral load suppression rate at 6 months, 12 months, and then monitoring every 12 months is an important clinical outcome, we did not analyse it because half of the patients were LTFU at six months. The viral load is analysed at six months to assess whether the patients are suppressed.

### Independent variables

Socio-demographic variables at enrolment in HIV care; including age, gender, marital status, and clinical data such as WHO clinical stage, CD4 cell count and the strategy for enrolment in HIV care were considered as potential predictors of LTFU.

### Data extraction

Data were collected from six facilities in Ekurhuleni north sub-district. The TIER.Net database was used to draw a list of records of the patients that were initiated on ART in November 2016. A sampling frame of records that met the inclusion criteria was created using unique patient´s identification numbers. The list was then used to pull out the patients´ paper medical records from the filing cabinets. Systematic sampling technique was used, until the number required from each facility was obtained. Records with missing data were excluded during data extraction. Data were extracted manually from individual patient records by the first author (PS) who is a trained health professional and understands both the TIER.Net and patient´s medical records. The TIER.Net generates variables such as the socio-demographics of the patients, ART start date, and the last visit date. All extraction was done on site; no records were removed from the filling room for confidentiality of patient´s records. In addition, only the record identify was extracted for purposes of anonymity.

### Data analysis

All statistical analyses were conducted using STATA 13.0 (StataCorp LLC, College Station TX). The primary outcome was LTFU and patients were grouped by ART initiation strategy (UTT and Pre-ART). We performed descriptive analyses for frequency and proportions of categorical variables and summary statistics for median and means for numerical variables. Clinical and categorical factors associated with loss to follow up at 12 months of ART were assessed by chi-square analysis. We performed multiple logistic regressions to identify predictors of LTFU up at 12 months of ART. We included variables that were associated with LFTU up in the bivariate analysis at p value of 0.05. We also included variables such as gender, age, and clinical data including CD4 count and WHO staging based on a documented association with loss to follow up in literature [25,39]. A p-value < 0.05 was considered statistically significant.

### Ethical considerations

Ethical clearance was obtained from the Ethics and Research Committee of Sefako Makgatho Health Sciences University (SMUREC/H/290/2017: PG). Permission to use patients´ records was obtained from Ekurhuleni Health District (Ref GP_201802_011) and from the operation managers of the selected facilities. As the study involved the use of data from the TIER.Net electronic database, patient consent was not required. To ensure the confidentiality of patients´ information, data extracted from the records was de-identified.

## Results

### Demographic and clinical characteristics of study sample

The sample comprised 367 records of patients enrolled into HIV care in the study setting. Across the sample, more patients were initiated through the UTT strategy compared to the pre-ART strategy, 198 (54%) vs. 169 (46%). More females 233 (63%) than males were initiated in care during the study period. The mean age at ART initiation was 36.3 years (SD 9.6; range, 18-72 years).The mean CD4 cell count at ART initiation was 341cells/mm^3^, 50% were initiated at CD4 cell count between 101 and 499 cells/mm^3^ and only 26% were initiated at CD4 cell count above 500 cells/mm^3^. The majority (79%) were classified as WHO stage 1, and more patients were initiated at WHO stage 1 through UTT than through pre-ART (81% vs. 77%).

Over two thirds (59.6%) of the patients were retained in care at 12 months, 28.4% were LTFU, and 12% were transferred out. More patients initiated through UTT were LTFU in care compared to those initiated through pre-ART (51% vs. 48.4%), but more patients were LTFU from the UTT cohort than the pre-ART (65.4% vs. 34.6%) was. All, the patients were initiated on first-line ART, which is a fixed dose combination of Tenofovir (TDF), Emtricitabine (FTC) and Efavirenz (EFV) (1TFE) Table 1.

**Table 1:** demographics and baseline clinical data of patients initiated on ART through UTT and Pre-ART in November 2016

Variable		Total	UTT (n=198)	Pre-ART (n=169)
**Gender**	Female	233 (63)	128 (65)	105 (62)
	Male	134 (37)	70 (35)	64 (38)
**Age category**	18-35 years	190 (51.2)	108 (53.2)	82 (48.8)
	36-45 years	115 (31)	63 (31)	52 (31)
	46-55 years	51 (13.8)	24 (24.8)	27 (16.1)
	56-72 years	15 (4.0)	8 (3.9)	7 (4.2)
**Marital status**	Single	234 (64)	117 (59)	105 (62)
	Married	133 (36)	69 (41)	64 (38)
**Employment status**	Employed	196 (52)	110 (56)	86 (52)
	Unemployed	171 (47)	88 (44)	83 (48)
**WHO staging**	WHO stage I	190 (79)	160 (81)	130 (77)
	WHO stage II	48 (13)	27 (14)	21 (12)
	WHO stage III	19 (5)	6 (3)	13 (8)
	WHO stage IV	10 (3)	5 (2)	5 (3)
**CD4 at initiation**	CD4 < 100	79 (21.3)	44 (21.7)	35 (20.8)
	CD4 > 500	94 (25.3)	42 (20.7)	52 (31)
	CD4 101-499	199 (53.5)	117 (57.6)	81 (48.2)
**Outcome at 12 months**	Retention in care	219 (59.6)	112 (51.6)	105 (48.4)
	Lost to follow up	104 (28.4)	68 (65.4)	36 (34.6)
	Transfer out	44 (12)	18 (47.7)	23 (52.3)

### WHO staging and CD4 count baseline characteristics

Table 2 presents the clinical data of patients. Compared with males, more females (81.4%) were initiated at baseline WHO stage I than males (75%). The mean CD4 cell count at ART initiation was 341 cells/μl. More males were initiated at baseline CD4 < 100 cells/μl compared to females (31.8% vs. 18.6%), and fewer males were initiated at baseline CD4 >500 cells/μl compared to females (16.3% vs. 31.6%). There was significant association between gender and CD4 count cells at initiation, males were less likely to be initiated at baseline CD4 >500 cells/μl compared to females (OR=0.35, CI: 0.20-0.61, p=0.001).

**Table 2:** baseline clinical characteristics of patients initiated on ART in November 2016 by gender (n=367)

	All	Females		Males		
		Freq.	Percent	Freq.	Percent	P value
WHO staging						
WHO stage I	292 (8.5)	193	81.4	99	75	0.230
WHO stage II	49 (13.2)	27	11.4	22	16.3	
WHO stage III	20 (5.4)	12	5.1	8	5.9	
WHO stage IV	11 (3)	5	2.1	6	4.4	
CD4 cell count at initiation						0.000
CD4< 100	79 (21.2)	44	18.6	42	31.8	
CD4 >500	94 (25.3)	75	31.6	22	16.3	
CD4 between 101-499	199 (53.5)	118	49.8	71	52.6	
ART Initiation strategy						
UTT	198 (54)	128	65	105	62	0.068
Pre ART	169 (46).	70	35	64	38	

### Characteristic of patients lost to follow up

Of the 104 patients that were LTFU, higher LTFU was observed for patients initiated through the UTT strategy 64.1% vs. 35.9% for pre-ART. The results also showed higher LFTU among females 69% vs. 31% for men, patients aged 18-35 than 46-61 years (54% vs 12%), those initiated with higher CD4 > 500 cell/mm^3^ than CD4 < 100 cell/mm^3^ (30% vs. 19%), and those with higher baseline WHO stage III than WHO stage I (84% vs. 6%). Almost half (49.5%) of patients were LTFU after the first six month of ART initiation, within the first three months, 28% of the patients who were LTFU had left care Table 3.

**Table 3:** socio-demographics and baseline characteristic of patients lost to follow up at 12 months

	Lost to follow up (n=104)		
	Variables	Freq./percent	P value
**Gender**	Males	32 (31)	0.187
	Females	70 (69)	
**Age category**	18-35 years	134 (56)	0.291
	36-45 years	80 (35)	
	46-55 years	42 (9)	
	56-72 years	12 (3)	
**WHO stage**	Stage I	6 (6)	0.102
	Stage II	10 (10)	
	Stage III	86 (84)	
	Stage IV	0	
**CD4 count at initiation**	CD4 < 100	19 (18.5)	0.378
	CD4 101-499	53 (51.5)	
	CD4 > 500	31 (30)	
**Time to LTFU**	Within 1 month	20 (19.4)	0.008
	1-3 months	9 (8.7)	
	4-6 months	22 (21.4)	
	7-11 months	52 (50.5)	
**ART initiation strategy**	Universal test and treat	66 (64.1)	0.025
	Pre-Art	37 (35.9)	

### Risks factor for LTFU inpatient initiated through UTT and Pre-ART

We assessed the time to LFTU and the CD4 count levels of patients found no significant differences in the CD4 counts of patients when they left care. Half of the patients who were LTFU left care after six months on ART and 50.6% had a baseline CD4 count of less than 100cell/mm^3^. Less than a quarter (19.4%) left care immediately after ART initiation, there was no significant difference in the baseline CD4 count for these patients Table 4.

**Table 4:** time to LTFU for patients initiated on ART through UTT and Pre-ART by CD4 count

Time to lost to follow up	Total	CD4 100-499	CD4 <500	CD4 >100
		Freq/Percent	Freq/Percent	Freq/Percent
Within 1^st^ month	20 (19.4)	27 (13.7)	12 (12.8)	11 (13.9)
1-3 months	9 (8.7)	27 (13.7)	21 (22.3)	14 (17.7)
4-6 months	22 (21.4)	64 (32.5)	18 (19.2)	14 (17.7)
7-11 months	52 (50.5)	79 (40.1)	43 (45.7)	40 (50.6)

### Factors associated with LFTU from pre-ART and UTT ART care

In the univariate analysis, we found a significant association between age (OR=0.64, CI: 0.42-0.97, p=0.037) and time to LTFU (OR=1.73, CI: 1.14-2.63, p=0.009) and initiation through UTT. Unexpectedly, more patients were less likely to be initiated with a baseline CD4 cell count above 500 cell/mm^3^ through UTT (OR=0.58, CI: 0.36-0.93, p=0.024). At multiple regression analysis, LTFU was associated with initiation through UTT (OR=1.84, CI: 1.13-3.00, p=0.025). There was no significant association between LFTU and gender, CD4 count, WHO staging, and age.

## Discussion

This study describes LTFU from HIV positive patients who were enrolled ART through the UTT and pre-ART strategies. Slightly more (54%) patients were initiated through UTT. A high proportion (79%) of the patients was asymptomatic at baseline and categorized into WHO clinical stage 1. The study was conducted during the launch of the UTT strategy where patients are initiated immediately after testing for HIV regardless of the CD4 count. Often this happens while the patients are still asymptomatic, this is the basis of the majority of patients presenting at WHO stage I.

The study found that 28% of all the patients LTFU at 12 months of ART. The rate of LTFU is consistent with rates reported in other studies in South Africa (25%) and Malawi (26%) among patient initiated through the UTT strategy [8,40]. The LFTU rate observed in this study is slightly lower than the 34% reported in a study of patients who initiated ART under the UTT strategy in Nigeria [31]. Of concern is that only 60% of the patients were retained in care. The retention in care at month 12, is lower than the target set by the South African National Strategic Plan (2017-2022), the estimated rate of 90% set by the South African Government, and international guidelines [6] needed to ensure the eradication of the HIV epidemic [40].

Half of the patients were LFTU within six months of ART, whilst 28% exited care within three months. A cluster-randomized trial in KwaZulu-Natal, in South Africa, reported that 31% of patients who exited care during the study period attended clinics only once [40]. Likewise, a recent treat all study in Nigeria reported that most LTFU occurred in the first 30 days after initiating treatment [31]. Early LTFU after ART initiation might be attributed to the fact that newly diagnosed persons who attend a clinic may not be ready to engage steadfastly in HIV care. Although there is overwhelming support for the UTT strategy, evident by the adoption of the strategy by most countries [35], the current study findings and those of others [31,40] suggest that a delay is required for individuals who test HIV positive to accept the diagnosis. There is need to provide psychosocial preparation and readiness assessment to newly diagnosed HIV-positive individuals before ART initiation under UTT [39]. This is necessary for individuals to make informed decision whether or not to attend a clinic.

Studies conducted under earlier treatment guidelines and the current UTT strategy indicated that CD4 count at ART initiation was associated with increased LTFU risk [25,31,39,40]. Although the current study did not find statistical significant association between CD4 count and LTFU, half of the patients LTFU after six months on ART, had a baseline CD4 count of less than 100cell/mm^3^. Lower baseline CD4 counts was associated with higher rates of attrition in other studies [34,41,42]. Research suggest that patients with low CD4 cell counts are likely to be in WHO stage 3 or 4, have opportunistic infections, and are too sick to present for follow-up or might have even died unbeknown to the health providers [7,43]. Other studies reported that patients starting ART at high CD4 count are at higher risk of LTFU because they might consider themselves as healthy [16,39,40].

The results further indicated that overall more females (69%) than males (31%) were LTFU but the difference was not statistically significant. The current study findings are in contrast with previous studies, Tweya *et al*. [8] found that being female was associated with a lower risk of LTFU, while several studies found that male gender was associated with an increased risk of LFTU [44,45]. Contrary to other studies who found that older age was associated with reduced risk of LTFU [8,37], in this study, age was not a risk factor for LTFU. This equally suggest that young age is a risk factor for LTFU [7,37,42,45] and that young people need to be supported through appropriate and effective strategies to stay in care [40].

The study found that LTFU was significantly associated with UTT, patients initiated through the UTT strategy had higher rates of LTFU than pre-ART patients had and were twice more likely to be LTFU (AOR=1.84, CI: 1.13-3.00). The results further showed that those initiated through the UTT strategy were almost twice likely to leave ART care within six months (OR=1.73, CI: 1.14-2.63) than those initiated through pre-ART. Intensive counsellor-driven interventions should address issues relating to retention in care for patients initiated on ART through UTT to retain patients in care. Additionally, implementation and strengthening of tracing strategies such as Short Message Service (SMS) reminders, phone calls and home visits by the tracing teams is an important public health intervention [39].

**Limitations:** the main limitation of this study was missing data, data were missing on VLD that limited the analysis, and discussion of viral load suppression measures for the patients that remained in care beyond six months. We acknowledge that some of the ART patients may have continued ART elsewhere without a formal transfer-out note, died or simply stopped ART.

## Conclusion

The study was conducted a year after the UTT strategy was launched which explains why only slightly over half of the patients were initiated ART through the UTT strategy. Over a quarter (28.4%) of the patients were LTFU and two-thirds (60%) were retained in care at 12 months of ART. The 12 months retention in care rate falls far short of the set targets for South Africa and globally.

### What is known about this topic

- Estimates of retention rate in patients initiated ART through the pre-ART strategy are widely variable and the estimated regional average 12-month retention of 76%;
- The triple 90 targets stipulate that 90% of all people receiving ART should be retained and have durable viral suppression.

### What this study adds

- Estimates of retention rate in patients initiated ART through the pre-ART and UTT strategies since studies conducted before the introduction of the UTT strategy cannot serve as indicators for LFTU in the context of UTT;
- Lost to follow up was associated with being initiated on ART through the UTT strategy;
- Patients initiated immediately after testing HIV positive may require additional visits to accept the HIV status before initiating ART.
